# Circulating Endothelial Progenitor Cells Decrease in Infants with Bronchopulmonary Dysplasia and Increase after Inhaled Nitric Oxide

**DOI:** 10.1371/journal.pone.0079060

**Published:** 2013-11-11

**Authors:** Yuanyuan Qi, Qian Jiang, Chao Chen, Yun Cao, Liling Qian

**Affiliations:** Departments of Pediatrics, Children’s Hospital of Fudan University, Shanghai, P. R. China; The Ohio State Unversity, United States of America

## Abstract

**Background:**

Impairment of endothelial progenitor cells (EPCs) has been shown to contribute to the development of bronchopulmonary dysplasia (BPD). In the current study, the relationship between EPC changes of after birth and the development of BPD was investigated, and the effects of inhaled nitric oxide (iNO) on EPCs were evaluated.

**Methods:**

Sixty infants with a gestational age of less than 32 weeks and a birth weight of less than 1500 g were studied. NO was administered to infants who were receiving mechanical ventilation or CPAP for at least 2 days between the ages of 7 and 21 days. EPC level was determined by flow cytometry at birth, 7, 21 and 28 days of age and 36 weeks’ postmenstrual age (PMA), before and after the iNO treatment. Plasma concentrations of vascular endothelial growth factor (VEGF), stromal cell-derived factor-1 and granulocyte-macrophage colony-stimulating factor were determined via immunochemical assay.

**Results:**

Twenty-five neonates developed BPD, 35 neonates survived and did not develop BPD. EPC level was decreased on day 7 and 21 in infants who later developed BPD compared with infants that did not develop BPD. From birth to 21 days of age, BPD infants had a persistently lower VEGF concentration compared with non-BPD infants. No difference was found between the two groups at day 28 or 36 weeks PMA. In infants that later developed BPD, iNO raised the KDR^+^CD133^+^ and CD34^+^KDR^+^CD133^+^ EPC numbers along with increasing the level of plasma VEGF.

**Conclusion:**

EPC level was reduced at 7 days of age in infants with BPD, and iNO increased the EPC number along with increasing the level of VEGF. Further studies are needed to elucidate the mechanism leading to the decrease of EPCs in infants with BPD and to investigate the role of iNO treatment in the prevention of BPD.

## Introduction

Bronchopulmonary dysplasia (BPD) is the main chronic respiratory disorder following premature birth and is diagnosed in approximately one quarter of infants less than 1500 g at birth [1]. Histologically, the “new BPD” is characterized by arrested lung growth, with decreased alveolarization and a dysmorphic vasculature [2]. Recent studies have suggested that blood vessels in the lung actively promote normal alveolar development [3], and that disruption of angiogenesis can impair alveolarization and may play a central role in the pathogenesis of BPD [4], [5].

Endothelial progenitor cells (EPCs) can migrate from bone marrow to the peripheral circulation, where they contribute to the repair of injured endothelium and to the formation of new blood vessels [6]. Several studies have suggested that EPCs may be involved in the pathogenesis of BPD. Balasubramaniam et al. [7] demonstrated that in neonatal mice, hyperoxia-induced EPCs reduction might contribute to impaired vascular and alveolar growth in the lung in cases of BPD. Recent studies have demonstrated that preterm infants, that subsequently develop BPD, have reduced numbers of EPC colonies in the cord blood [8], [9]. These results suggest that a decrease in EPCs at birth may contribute to the development of BPD. In contrast, a different clinical study reported that the number of EPCs present in peripheral blood at birth did not appear to affect the risk of developing BPD [10]. The contrasting results may reflect the fact that the number of EPCs in cord blood does not completely equate to the number present in the peripheral blood due to the impact of many factors that take place after birth. Therefore, the EPC level in peripheral blood may better predict the risk of developing BPD in preterm infants. However, dynamic changes in infant EPC levels after birth and the association with the development of BPD due to these changes has not been well documented.

In recent years, many studies have focused on the role of inhaled nitric oxide (iNO) for the prevention and treatment of BPD. A number of multicenter clinical trials have shown that iNO therapy reduces the incidence of BPD [11]–[13]. However, Mercier JC et al demonstrated that early use of iNO had not reduced the incidence of BPD in preterm infants [14]. The exact role of iNO and its mechanisms are still unclear. Studies in animal models have suggested that iNO stimulates angiogenesis and alveolarization [15] through enhanced signaling mediated by VEGF [16], [17]. Moreover, studies have demonstrated that endothelial nitric oxide synthase (eNOS) and NO play an important role in EPC mobilization [18], [19]. ENOS transcription enhancer and NO donor supplementation could increase the EPC level and improve EPC function [20], [21]. A recent *in vitro* study reported that NO gas treatment, during hyperoxia exposure, increased the growth of preterm cord blood-derived EPCs [22]. Moreover, in a previous study, we found that iNO promoted the mobilization and release of EPCs from bone marrow into circulation and contributed to vascular repair in a piglet model of acute lung injury [23]. However, it remains unknown whether iNO increases the EPC level, thus preventing the development of BPD, in premature infants.

The aim of this study was to investigate the dynamic changes of EPCs after birth and to evaluate the effects of iNO on peripheral EPC number and on the prevention and/or treatment of BPD.

## Methods

### Ethics Statement

The Ethics Committees of Children’s Hospital of Fudan University approved this study (Approval Number: 2010003). Written informed consent was obtained from the parents of infants prior to NO inhalation and blood collection.

### Study Design

Preterm infants with a gestational age of less than 32 weeks and a birth weight of less than 1500 g, were included in the study. All infants had been admitted within the first 6 hours of life to the tertiary neonatal intensive care unit between October 2010 and June 2011. Infants with congenital anomalies were excluded from the study. The infants were followed up until 36 weeks’ postmenstrual age (PMA). At the same time, the initiation of iNO was recorded. The iNO treatment was given to infants according to physician’s experience and infant’s condition, especially for infants who were receiving assisted ventilation for lung disease (not apnea) between 7 and 21 days [11]. The inhaled NO was administered into the inspiratory limb of the ventilator circuit or between the Infant Flow driver and a humidifier using an iNO delivery system (Noventek, Shanghai, China). The NO and nitrogen dioxide levels at the end of the inspiratory limb were continuously monitored using electrochemical monitors (NOxBOX, Bedfont Ltd, UK). INO was given at 5 ppm for one day and then decreased to 2 ppm until weaning. INO treatment was administered for a minimum of 7 days.

### Data Collection

Clinical and outcome data were prospectively collected from the study population. The Clinical Risk Index for Babies-II score (CRIB-II) for the prediction of neonatal mortality and morbidity was calculated. The cumulative oxygen exposure value was calculated as reported previously [24]. BPD was defined as oxygen dependence for at least 28 postnatal days [25]. The severity of BPD was graded as mild, moderate or severe according to Jobe and Bancalari [26]. The association of EPC number at different time points with the development of secondary outcomes such as, patent ductus arteriosus (PDA), necrotizing enterocolitis (NEC), retinopathy of prematurity (ROP), intraventricular hemorrhage (IVH) and periventricular leukomalacia (PVL) were also examined.

### Sample Collection

Peripheral blood samples were collected during routine laboratory exams at the following time points: birth (within the first 12 h of life), 7 days, 21 days, 28 days of age and 36 weeks’ PMA. Blood samples were also obtained in infants who received iNO therapy before the initial iNO treatment and again after 7 days of inhalation. Blood samples were collected in heparinized tubes. About 0.1 ml of fresh whole blood was used for flow cytometry. Plasma was collected and stored at −20°C for further analysis.

### Flow Cytometry

Cells were assessed in fresh whole blood samples as previously described [23], using flow cytometry on. Briefly, 100 ul of peripheral blood was incubated with 10 ul FITC-conjugated CD34 monoclonal antibodies (mAbs) (BD Biosciences, CA, USA), 5 ml of APC-conjugated CD133 mAb (Meltenyi Biotec, CA, USA), and 10 ml of PE-conjugated KDR mAb (R&D Systems, Minneapolis, MN, USA). Blood was then stored at 4°C for 30 min. Red cells were then lysed and the frequency of EPCs was determined by a two-dimensional forward scatter/fluorescence dot plot analysis of the samples (FACSAria; BD Biosciences, Franklin Lakes, NJ, USA). Isotype controls were used for setting gates and for determining the positive/negative boundaries. The analysis was confirmed by running the FMO (fluorescence-minus-one) controls. After morphological gating was done to exclude granulocyte and cell debris, CD34^+^ or CD133^+^ cells were gated and the resulting population was examined for dual expression of KDR, and triple positive cells were identified by the dual expression of KDR and CD133 in the CD34 gate. Approximately 500,000 cells were acquired. The EPC levels were expressed as the percentage of total mononuclear cells (MNC). The same trained operator performed all the tests throughout the study and was blind to the experiment.

### Enzyme-linked Immunosorbent Assay

Plasma levels of VEGF, granulocyte-macrophage colony-stimulating factor (GM-CSF), stromal cell-derived factor-1 (SDF-1) and eNOS (R&D Systems, Minneapolis, MN, USA) were assessed using enzyme-linked immunosorbent assay kits. The procedures were performed according to the manufacturer’s instructions.

### NO Assay

NO concentration in plasma was evaluated by measuring the total NO_2_^−/^NO_3_^−^ using the Griess reaction (Jiancheng Bioengineering Institute, Nanjing, China).

### Statistical Analysis

The parametric and nonparametric continuous variables are reported as mean ± standard deviation (SD) and the median and interquartile range or range, respectively. Categorical variables are described by count and relative frequency (%). Comparison between groups of continuous parametric and nonparametric variables was carried out using the Student’s t-test and the nonparametric Mann-Whitney U-test, respectively. Categorical variables were compared between groups using the Fisher’s exact test. A *P* value <0.05 was considered statistically significant.

## Results

### Characteristics of Infants

Of the 60 infants that were included in the study, 25 developed BPD, 35 survived without BPD. The clinical characteristics, including maternal data and incidence of complications are displayed in Table 1. The mean gestational age (GA) and birth weight were lower in infants who developed BPD compared with infants who did not. BPD infants had significantly higher CRIB II scores. There was no difference in the premature rupture of membranes (PROM) when comparing infants who later developed BPD to infants that did not.

**Table 1:** Maternal, neonatal characteristics and outcomes of the study infants.

Characteristic	All (n = 60)	BPD (n = 25)	No BPD (n = 35)	*P* value
GA, mean ± SD, weeks	29.5±1.7	28.3±1.7	30.3±1.0	<0.001
BW, mean ± SD, g	1285±256	1136±209	1394±241	<0.001
Mode of delivery, cesarean/vaginal	34(56.7)/26 (43.3)	10 (40)/15 (60)	24 (68.6)/11 (31.4)	0.036
Gestational diabetes mellitus, n (%)	6 (10)	2 (8)	4 (10.5)	1.0
Maternal hypertension, n (%)	11 (18.3)	3 (12)	8 (22.9)	0.332
Premature rupture of placenta>24 h, n (%)	10 (16.7)	4 (16)	6 (17.1)	1.0
Antenatal steroids, n (%)	33 (55)	11 (44)	22 (62.9)	0.191
Sex, male/female, n (%)	34 (56.7)/26 (43.3)	13 (52)/12 (48)	21 (60)/14 (40)	0.603
SGA, n (%)	7 (11.7)	3 (12)	4 (11.4)	1.0
Apgar-5, median (range)	8 (3–10)	7 (3–10)	9 (5–10)	0.003
CRIBII, median (range)	6 (2–14)	8 (4–14)	5 (2–13)	<0.001
PDA, n (%)	33 (55)	19 (76)	14 (40)	0.008
NEC, n (%)	6 (10)	4 (16)	2 (5.7)	0.223
IVH,I–IV, n (%)	12 (20)	6 (24)	6 (17.1)	0.122
PVL, n (%)	10 (16.7)	7 (28)	3 (8.6)	0.077
ROP, n (%)	13 (21.7)	9 (36)	4 (11.4)	0.03
Postnatal steroids, n (%)	2 (3.3)	2 (8)	0 (0)	0.161
PS, n (%)	43 (71.7)	21 (84)	22 (62.9)	0.089
Length of hospital stay, median (range), d	51 (21–192)	65 (29–192)	45 (21–78)	0.031
Oxygen, median (range), d	9.9 (0–51.5)	28.8 (23.4–51.5)	5.0 (0–21)	<0.001
Oxygen AUC, median (range)	1218 (0–13247)	4305(1097–16606)	610 (0–5374)	<0.001
MV, median (range), d	3.4(0–44.1)	18.4 (0–44)	0 (0–15.3)	0.001
nCPAP, median (range), d	3.8(0–42)	17.5 (3.8–38)	1.6 (0–19.7)	0.000
Cost of hospital, median (range), ×10^3^ CNY	4.5(1.2–28.6)	9.1 (6.3–28.7)	3.7 (1.2–9.5)	0.000

BPD = bronchopulmonary dysplasia; CRIB-II = Clinical Risk Index for Babies-II score; PDA = patent ductus arteriosus; NEC = necrotizing enterocolitis; IVH = intraventricular hemorrhage; PVL = cystic periventricular leukomalacia; ROP = retinopathy of prematurity; PS = pulmonary surfactant; MV = mechanical ventilation; nCPAP = nasal continuous positive airway pressure; CNY = Chinese Yuan.

Infants with BPD had a longer duration of exposure to oxygen, nasal continuous positive airway pressure and mechanical ventilation. The cumulative oxygen exposure value was significantly higher for infants who developed BPD compared with those who did not. The incidence of patent ductus arteriosus (PDA) and ROP was significantly higher in infants who developed BPD vs. those that did not.

Of the infants who developed BPD, 20 infants received iNO therapy, with a mean GA of 28.1±1.3 weeks, and a mean BW of 1166±198 g. The starting time of the NO treatment was 17.0±8.1 days of age.

### The Dynamic Changes of EPCs in Infants with and without BPD

There was no difference in the WBC counts at birth between infants who subsequently developed BPD and those who did not (9.3±6.5 vs. 12.7±8.1 ×10^9^ per L, *P* = 0.153). EPC levels at different time points after birth were compared. In both the BPD and non-BPD groups, the number of CD34^+^ cells were highest at birth, but were markedly decreased by day 7 (*P* = 0.003). There was a slight trend for CD34^+^ and CD133^+^ cells to be decreased during different time points after day 7, although this was not significant.

There was no difference in all of the EPC sub-types at birth when comparing infants with and without BPD. However, the percentage of CD34^+^KDR^+^, KDR^+^CD133^+^ and CD34^+^KDR^+^CD133^+^ cells was significantly lower on day 7 in infants that developed BPD than in those without BPD (Table 2). Moreover, on day 21, the level of CD34^+^KDR^+^CD133^+^ cells was lower in infants with BPD than in those without BPD. Also, there was a trend for the number of CD34^+^KDR^+^ and KDR^+^CD133^+^ cells to be lower in BPD infants compared to infants without BPD. No difference was found between the 2 groups on day 28 or at 36 weeks’ PMA.

**Table 2:** The percentages of cell subsets in circulating blood at different time points in infants with and without BPD.

	Group	D 0	7 d	21 d	28 d	36 w
CD34^+^/MNC, %	BPD	0.757(0.481–1.36)	0.434(0.230–0.729)	0.419(0.279–0.653)	0.357(0.227–0.629)	0.331(0.131–0.500)
	No BPD	0.654(0.268–1.24)	0.317(0.207–0.486)	0.324(0.172–0.631)	0.341(0.220–0.625)	0.279(0.145–0.431)
	*P*	0.441	0.144	0.327	0.782	0.912
CD34^+^KDR^+^/MNC, %	BPD	0.023(0.010–0.094)	0.020(0.009–0.026)	0.015(0.008–0.024)	0.016(0.007–0.028)	0.017(0.008–0.035)
	No BPD	0.034(0.012–0.056)	0.026(0.013–0.042)	0.022(0.012–0.030)	0.016(0.010–0.030)	0.017(0.012–0.030)
	*P*	0.831	0.026	0.060	0.414	0.956
KDR^+^CD133^+^/MNC, %	BPD	0.006(0.002–0.009)	0.004(0–0.015)	0.003(0.001–0.005)	0.004(0.002–0.010)	0.005(0.002–0.016)
	No BPD	0.009(0.004–0.016)	0.008(0.004–0.013)	0.005(0.003–0.009)	0.004(0.002–0.006)	0.005(0.003–0.010)
	*P*	0.190	0.004	0.072	0.406	0.982
CD34^+^KDR^+^CD133^+^/MNC, %	BPD	0.005(0.002–0.009)	0.004(0.002–0.007)	0.002(0.001–0.005)	0.004(0.002–0.010)	0.005(0.002–0.015)
	No BPD	0.007(0.004–0.015)	0.007(0.004–0.012)	0.004(0.003–0.010)	0.004(0.002–0.005)	0.006(0.003–0.009)
	*P*	0.190	0.023	0.038	0.250	0.982

Data are presented as medians with 25–75% quartiles. MNC = mononuclear cells.

Interestingly, we found that BPD infants had a persistently lower VEGF concentration compared with the non-BPD infants from birth to day 21 (Table 3). No difference was found in plasma SDF-1 levels between infants with and those without BPD at any time point, although SDF-1 levels tended to be lower in BPD infants. There was no significant difference in the level of plasma GM-CSF at any time point between infants with and without BPD. Unexpectedly, we found that NO level was decreased in BPD infants compared to non-BPD infants on day 7 and 28.

**Table 3:** The levels of plasma cytokines at different time points in infants with and without BPD.

	Group	D 0	7 d	21 d	28 d
VEGF(pg/ml)	BPD	178.2±156.4	394.3±127.9	287.9±163.9	512.8±202.8
	No BPD	671.3±441	727.4±441.9	638.5±412.5	534.3±307.4
	*P*	0.032	0.005	0.042	0.895
SDF-1(ng/ml)	BPD	2.0±1.2	2.5±0.9	2.9±0.8	2.6±0.6
	No BPD	3.2±0.8	3.3±0.4	3.6±0.8	3.0±0.8
	*P*	0.101	0.191	0.168	0.268
GM-CSF(ng/ml)	BPD	1.6±1.5	2.6±1.9	2.5±1.3	1.9±1.1
	No BPD	3.0±0.5	5.0±1.3	4.0±2.5	3.2±2.9
	*P*	0.192	0.085	0.136	0.070
NO_2_^−/^NO_3_^−^(umol/l)	BPD	34.4±29.9	29.5±19.4	33.7±22.6	30.4±23.7
	No BPD	38.8±36.6	46.7±36.1	48.7±35.5	48.5±25.2
	*P*	0.708	0.050	0.147	0.004

VEGF = vascular endothelial growth factor; SDF-1 =  stromal cell-derived factor-1; GM-CSF = granulocyte-macrophage colony-stimulating factor. Data are presented as mean ± SD.

**Figure 1:**
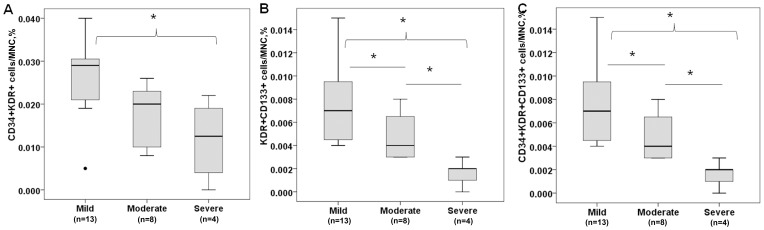
EPC level in infants with different severity of BPD at day 7. (A) CD34^+^KDR^+^ cells. (B) KDR^+^CD133^+^ cells. (C) CD34^+^KDR^+^CD133^+^ cells. The level of EPCs was significantly lower in infants with severe BPD compared to infants with mild BPD. Values in boxplot are expressed as median, 25th, and 75th percentiles. MNC =  mononuclear cells. *P<0.05.

In addition, the association between the EPC level and the severity of BPD was evaluated. Severe BPD infants displayed lower levels of EPCs compared to mild BPD infants on day 7, which was statistically significant for KDR^+^CD133^+^ cells (0.002[0.001–0.002]% vs. 0.007[0.005–0.022]%; P<0.01) and CD34^+^KDR^+^CD133^+^ cells (0.002[0.001–0.002]% vs. 0.007[0.005–0.020] %; P<0.01) (Fig. 1). There was no difference in EPC subtypes between infants with mild, moderate or severe BPD infant was observed at birth or at any other time points.

### The EPC Number Increased after iNO in BPD Infants

**Figure 2:**
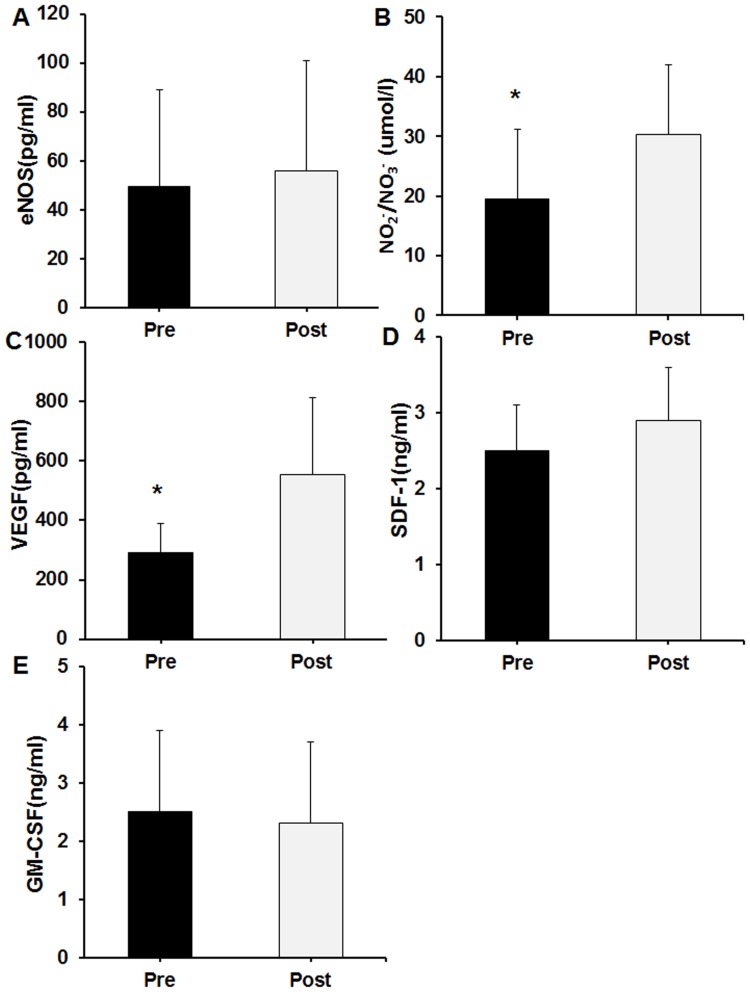
Plasma cytokines levels before and after iNO therapy. (A) eNOS. (B) NO_2_^−/^NO_3_^−^. (C) VEGF. (D) SDF-1. (E) GM-CSF. Pre represents before iNO treatment, and Post represents after iNO treatment. eNOS = endothelial nitric oxide synthase; VEGF = vascular endothelial growth factor; SDF-1 =  stromal cell-derived factor-1; GM-CSF = granulocyte-macrophage colony-stimulating factor. *P<0.05.

There was no difference when comparing the WBC count before and after iNO therapy (11.8±6.1 vs. 10.1±5.2 ×10^9^ per L, *P*>0.05). However, the number of KDR^+^CD133^+^ and CD34^+^KDR^+^CD133^+^ increased after iNO therapy (Table 4). Other cell populations other (CD34^+^, CD133^+^, CD34^+^CD133^+^, and CD34^+^KDR^+^ cells) also tended to increase after the iNO therapy. Interestingly, inhaled NO also increased the plasma VEGF (289.7±101.2 vs. 554.5±259.7 pg/ml, *P*<0.05) and NO (19.5±11.6 vs. 30.2±11.7 umol/l, *P*<0.05) concentrations (Fig. 2). There was no difference between pre- and post- iNO treatment levels of eNOS, SDF-1 and GM-CSF.

**Table 4:** The percentages of cell subsets in circulating blood pre and post iNO therapy.

	Before iNO	After iNO	*P* value
CD34^+^/MNC, %	0.331(0.215–0.935)	0.391(0.234–0.605)	0.964
CD34^+^KDR^+^/MNC, %	0.022(0.012–0.033)	0.026(0.016–0.046)	0.121
KDR^+^CD133^+^/MNC, %	0.004(0.002–0.008)	0.008(0.004–0.030)	0.014
CD34^+^KDR^+^CD133^+^/MNC, %	0.004(0.002–0.007)	0.007(0.005–0.024)	0.011

Data are presented as medians with 25–75% quartiles. MNC = mononuclear cells.

### The Relationship between EPC Level and Secondary Outcomes

We also assessed whether different cell phenotypes were correlated with the presence of other diseases in preterm infants. We found that infants who later developed ROP tended to have higher levels of circulating progenitor cells at birth than infants who did not, which was statistically significant for CD34^+^ (2.95[0.784–4.18]% vs. 0.746[0.10–3.99]%, *P* = 0.017), CD133^+^ (1.89[0.472–2.71]% vs. 0.548[0.072–2.39]%, *P* = 0.044), and CD34^+^CD133^+^ cells (1.65[0.443–2.40]% vs. 0.470[0.053–2.17]%, *P* = 0.029). However, there were no significant differences in the concentration of VEGF between the two groups (584.2±244.5 pg/ml vs. 480.3±269.7 pg/ml; *P* = 0.088). Conversely, no differences were observed between infants with and without IVH, PDA and NEC in any of the studied cell subsets studied.

## Discussion

The contribution of impaired vascular growth to the development of BPD has been extensively studied. Several studies have demonstrated that EPCs may contribute to angiogenesis in the developing lung and have shown promise for the prevention and treatment of BPD. In the current study, we found that infants with lower EPC levels during early days after birth had higher risk of developing BPD. Furthermore, we found that iNO treatment could increase the level of circulating EPCs.

It should be acknowledged that no consensus has yet been reached regarding methods for EPC identification. Flow cytometry is a convenient, rapid, and purely quantitative method used for population based studies. In many studies, three surface antigens and the corresponding subpopulations of EPCs (CD34^+^KDR^+^, KDR^+^CD133^+^ and CD34^+^KDR^+^CD133^+^ cells) have been used for EPC enumeration, and have been shown to correlate with the severity of the disease state [27]–[29]. In this context, we studied these specific combinations of antigens to detect different EPC populations and their correlation with clinical and experimental data.

Recent studies have reported somewhat contradictory results as to whether EPC number is related to the development of BPD. A previous study using flow cytometry showed that infants who had a lower number of EPC colonies in cord blood and circulating EPCs at birth were at an increased risk of developing BPD [8]. However, other studies that used flow cytometry for EPCs quantification reported no association between EPC levels at birth and the development of BPD [10]. In contrast, a recent study demonstrated that a high number of circulating EPCs at birth was associated with higher risk of BPD [30], however, this study did not rule out the potential influence of GA. In the present study, we did not find a lower level of EPCs in infants who later developed BPD at birth. This could be attributed to the lower GA in the group that later developed BPD since an inverse correlation between EPC and GA has been well documented, especially during the first 48 h of life [31]. We did find that the EPC level was significantly decreased on day 7 in infants that later developed BPD compared to infants without BPD. And this has not been reported previously. These data suggested that a decreased EPC level during the first week of life might be an important factor responsible for the development of BPD in preterm infants. Moreover, we found that infants with severe BPD had the lowest EPC level on day 7. Based on this finding, we propose that the EPC level is negatively correlated with the severity of BPD, indicating that EPCs play a role in the lung vessel growth in premature infants. Circulating EPC level may serve as an indicator for the risk of BPD, however, the exact effects of EPCs on blood vessel growth and on the development of BPD are still unclear.

In the study, we report a higher incidence of BPD. This in an observational study instead of a randomized control trial, the iNO treatment depends on the clinical factors rather than randomize to iNO therapy according to the inclusion criteria. This might result in the more severe degree of lung condition and higher incidence of BPD in iNO group.

It has been recognized that VEGF signaling plays a crucial role during alveolar development in early postnatal life [32], [33]. Blockade of VEGF receptor activity during neonatal period impairs lung structure, similar to lung histology found in BPD [34], [35]. Moreover, VEGF is an important mediator of EPC mobilization [36]. We found that plasma VEGF is persistently reduced from birth to 21 days of age in infants with BPD compared to those without BPD. We speculate that the reduction in VEGF results in a decreased level of EPCs leading to the impairment of lung vascular growth. This observation requires further investigation since the precise mechanism of VEGF impairment is still unknown.

No difference was found in SDF-1 and GM-CSF, though both have been reported to be involved in the EPC mobilization [37], [38]. This difference may be attributed to the different pathobiology of neonatal lungs and to hyperoxia damage. The actual mechanisms by which VEGF mediates the mobilization of EPCs, under conditions such as premature birth, remain unknown.

In recent years, inhaled NO has been used as prophylactic treatment for the prevention of BPD. Experimental studies have suggested that iNO treatment, after neonatal VEGF receptor inhibition, preserves normal alveolar and vascular growth and reduces endothelium apoptosis [17], [39]. A previous study done by our group also showed that NO inhalation promote EPC mobilization and contribute to lung injury repair in the oleic acid induced piglet ARDS model [23]. In the current study, we found that iNO raised the number of circulating EPCs in infants after treatment. To our knowledge, this is the first study to demonstrate that inhaled NO induced an increase in EPC levels in preterm infants. These results suggest that inhaled NO may prevent BPD by increasing the circulating EPC level. Furthermore, the increase in EPCs, after iNO treatment, was associated with an increase in plasma VEGF. One of the downstream effects of VEGF signaling is the activation of eNOS and generation of NO [40], [41], which is an important factor for EPC mobilization from bone marrow. We speculate that iNO may be effective in BPD through the VEGF signal. However, this is a pilot observational study and we did not evaluate the effects of iNO on the incidence of BPD regarding VEGF. The exact mechanism by which iNO increases VEGF and mobilizes EPCs is still unclear and warrants further investigation.

We did not observe a difference in EPCs between infants with and without PDA, though this finding is not incompatible with a previous study [10] and, may also be due to the impact of GA. Nevertheless, the precise contribution of circulating EPCs to vessel growth is not completely understood.

In conclusion, we found a decrease in the EPC level on day 7 in infants who later developed BPD compared to infants that did not develop BPD. We also found that iNO raised the circulating EPC level along with the level of plasma VEGF. Further studies are needed to elucidate the mechanism for the development of BPD and to clarify the effects of VEGF signal regulation and circulating EPC alteration.
